# Supplementary material for: Effect of adjuvant radiotherapy in elderly patients with breast cancer
Source: PLoS One. 2020 May 20;15(5):e0229518. doi: 10.1371/journal.pone.0229518 (PMC7239665; doi:10.1371/journal.pone.0229518)
Supplement: Attachment — Submitted filename: Responsetoreviewers.docx [file pone.0229518.s001.docx]

**Response to the Reviewers**

**Additional Editor comments:**

**Major points:
-The intrinsic types of breast cancer are not defined according to prior studies (PMID: 29132568).**

*Thank you for this comment. As we mentioned in the section “Surrogate Definition of intrinsic subtypes” expression of hormone receptors, HER2 and analysis of Ki67 is generally used to define the intrinsic subtypes in breast cancer. Unfortunately, Ki67 was not available in our database. Therefore, the distinction between Luminal A and B was a challenge. Based on the publications of Minckwitz et al., Parise et al. and Lips et al. we used grading as a surrogate parameter for Ki67 as we did in all our previous BRENDA publications.*

*Anyway, the analysis and interpretation of the marker Ki67 is complex as some studies have used 10%, 14% or 20% as cut-off value. This problem is also discussed in PMID: 29132568 you mentioned.*

**-Fisher exact test should be used in some subgroups' analyses, instead of Chi-square test.**

*As we all know, the usual rule of thumb for deciding whether the chi-squared approximation is good enough is that the chi-squared test is not suitable when the* ***expected values*** *in any of the cells of a contingency table are below 5, or below 10 when there is only one degree of freedom. In our case we have no cells with an expected frequency of less than 5. Therefore, the chi-square test is good enough.*

*Nevertheless, we additionally calculated Fisher’s exact test beside of two other tests (likelihood ratio and linear by linear association).*

*The asymptotic significance of the chi-square test and the exact significance of Fisher’s exact test are in our case identical at least to 3 decimal places. Therefore, we renamed “significance” in table 1 “basic characteristics” to “asymptotic and exact significance”.*

*Here are the results*

**Age groups**

**T categories**

**Grading**

**Nodal status**

**Intrinsic subtypes**

**100% guideline adherence**

**Guideline adherent breast conserving therapy (BCT)**

**Guideline adherent radiotherapy**

**Adjuvant systemic therapy (AST)**

**-Please compare the characteristics of the subgroups that were generated by PSM-IPW grouping. I.e. to test whether PSM-IPW worked.**

*Thank you for this very interesting comment. We compared the characteristics of the subgroups that were generated by PSM-IPW grouping and indeed, in our case PSM-IPW increases the imbalance between the groups. This is just what Gary King and Richard Nielsen (2018) stated in their publication “Why Propensity Scores Should Not Be Used for Matching” (although propensity score matching is an enormously popular method of preprocessing data for causal inference) [27]. They showed that “PSM often accomplishes the opposite of its intended goal — thus increasing imbalance, inefficiency, model dependence, and bias. The weakness of PSM comes from its attempts to approximate a completely randomized experiment, rather than, as with other matching methods, a more efficient fully blocked randomized experiment. PSM is thus uniquely blind to the often large portion of imbalance that can be eliminated by approximating full blocking with other matching methods.”*

*Therefore, we no longer use PSM-IPW. We test now if there is a difference between two or more survival curves using the G^p^ family of tests of Harrington and Fleming (1982), with weights on each death of S(t)^p^, where S(t) is the Kaplan-Meier estimate of survival. With
rho = 0 this is the log-rank or Mantel-Haenszel test, and with rho = 1 it is equivalent to the Peto & Peto modification of the Gehan-Wilcoxon test. As result, there is no significant difference (p=0.7) between the survival curves of low risk patients with radiation and those without radiation.*

**-How many of your patients probably have used gene based biomarkers (Oncotype or mammoprint)? As recent trials show, they may influenced the patient treatment and outcomes.**

*At the time of data collection between 2001 and 2009 multigene expression assays were not the standard of care. So, for this retrospective analysis we do not know if individual patients received biomarker testing. But we agree that for further studies there will be subgroups of patients in which the multi gene expression assays will provide further helpful information and will also influence the patient`s decision on treatment strategy.*  **Minor points:
-Please cite related articles (PMID: 29132568, PMID: 28746732 and PMID: 31451977)**

*We cited the related articles*

**-Please indicate in the main text, not in the supplement, that the ethical approval has been reviewed and obtained.**

*We indicated that ethical approval has been obtained in the main text.*

**Reviewer #1: You state” The treatment of elderly breast cancer patients differs from the therapeutic approach in younger ones, as elderly patients are prone to geriatric frailty and comorbidities, such as renal failure, 94 liver disease, and/or cerebrovascular disease (3-5).”
Please add that the treatment also changes because the cancers are less aggressive (Even within ER +, elderly seem more responsive to endocrine therapy as the percent ER positivity is higher.)**

*Thank you for this important comment. We added in the discussion section that therapeutic strategies in elderly patients often differ from those of younger ones due to less aggressive tumor types.*
 **You over state the benefits of RT:
“After evaluation of different subtypes of breast cancer, it is obvious that even those with less aggressive molecular types profit from radiotherapy. In different meta analyses, hazard ratios of 0.2-0.35 have been described meaning that up to eight of ten relapses can be avoided by RT (6-8). As a result of the improved local tumor control, it has been previously stated that breast cancer related mortality has been significantly decreased during the last decades (6). Therefore, it has been a general recommendation to perform BCT only in combination with postoperative radiotherapy (9). If patients cannot or will not accept postoperative RT, mastectomy is the alternative surgical treatment (10). However, subgroups have been defined that are associated with a low risk for recurrence (pT1, pN0, R0, HER2-) (11, 12). In those cases, radiotherapy still adds benefits but can most likely be dispensed even after BCT.”
No randomized trial has shown a survival benefit to RT in early breast cancer.**

*In the cited meta-analysis by the EBCTCG* *17 randomized trials were analyzed and showed that RT to the breast reduced the breast cancer death rate by about a sixth.*

**Always state “locoregional” recurrence, as RT has no impact on distant recurrence in randomized trials.**

*Thank you for this comment. We have changed the section to make clear that we talk about locoregional recurrence.* **You never defined intermediate vs high risk or separated out those groups. These need to be defined.**

*In fact, we revised the manuscript and only have 2 groups now: low-risk patients who do not need additional radiation therapy and patients at higher-risk who seem to need radiation therapy.*

**You combine intermediate and high risk into a single group without showing that they act similarly. You need to describe why you made this combination.** *When we analyzed our data we found that the major differences occurred between low-risk patients and others. As mentioned above we no longer differentiate between intermediate and high risk patients.*

**In some places you state high risk benefits and in others that intermediate or high risk benefits, but I do not see these differentiated in your data.**

*Thank you for this comment. We adjusted the article accordingly. We no longer differentiate between intermediate and high risk patients, instead we use the term higher risk.*
 **Your intermediate group likely contains patients that fit the randomized trials that showed RT has not beneficial. (Such as a 1 cm Grade 3 cancer.). If you are using retrospective data to invalidate the conclusions of a randomized trial, please state why you think this is defensible.**

*We would really like to correct this issue since* *we don`t try to use retrospective data to invalidate the conclusions of the randomized trial CALGB 9343. Hughes et al. included patients >69 years with T1N0 ER+ breast cancer only. In this trial they did not differentiate between intrinsic subtypes. They found a small decrease in IBTR but no significant benefit in survival.*

**When you state that limiting your analysis to 9343 criteria, are you including intermediate and high risk patients who meet that criteria? If so, does that negate your statement that intermediate and high risk need RT?**

*Thank you for this interesting comment. A similar question can be asked for CALGB 9343. A better way to think of risk is as the possibility or probability of recurrence or death. Therefore, patients at higher risk without RT have a higher probability of recurrence or death compared to low risk patients. Nevertheless, there are patients at higher risk without RT, who have no recurrent disease. In the CALGB 9343 trial, as well as this study two types of low risk patients are defined, which both don´t need RT. Therefore, we can combine these two subsets to a new subset of low risk patients. For this combined subgroup we have no significant difference in recurrence free survival (log rank (Mantel-Cox) p=0.958) and tumor associated survival (log rank p= 0.506)*

**Reviewer #2: The manuscript here is technically sound and adds additional important information to the literature using a relatively large database of elderly women with breast cancer. The manuscript could be simpler and clearer and my suggestions are below:
1. The use of the terms BCT with and without RT is confusing. If I’m not mistaken breast conserving therapy without radiotherapy should be referred to as BCS (breast conserving surgery). BCT implies the use of radiotherapy.**

*Thank you very much for this important issue. We have revised the terms.*

**2. I generally disagree with intro lines 102-104. There has been enough data on low risk patients with small tumors having low recurrence rates without radiotherapy that these statements do not apply in 2019.**

*Thank you very much for this comment. We have clarified the introduction.*

**3. I’m not sure why the authors separated the groups out by low and intermediate/high. They define the patients that they place in this intermediate/high group but never define the difference between intermediate and high. This should be defined at some point.**

*Since reviewer 1 discussed the same issue we would kindly refer to the statement further above.*

**4. There are a great deal of words in the paragraph called “basic characteristics” and it is a difficult read. It is essentially explaining Table 1. The paragraph could be started by simply stating that “Basic patient characteristics are listed in Table 1” and then just point out a couple of important distinctions. I do not feel this large paragraph is necessary.**

*We have revised the section in order to shorten the paragraph.*

**5. I find the terms throughout the manuscript of guideline adherent and guideline non-adherent confusing. One could simply state that guidelines state that radiotherapy is indicated and compare the difference between those having it and those not having it. It also makes the Figures showing relapse free and overall survival confusing.**

*Thank you for this comment. We think that it is quite interesting to see in how many cases RT was not carried out even though the national guidelines would have recommended to do (at least 27,3 %!). Around 30% of those were of intermediate- and high-risk tumors and we showed that those patients profited from RT.*

**6. Discussion could be much simpler. Prime 2 and CALGB trials are referenced early on and then a paragraph is allotted to each one later in the discussion. I would just acknowledge each randomized control trial once and shorten discussion.**

*We adjusted the discussion according your recommendation.*

**7. Overall useful information particularly regarding the higher risk group but could be more simply and clearly presented.**

*We tried to reword the manuscript in order to achieve a clearer presentation.*
